# Tuning the Electrochemical Properties of Organic Battery Cathode Materials: Insights from Evolutionary Algorithm DFT Calculations

**DOI:** 10.1002/cssc.201903450

**Published:** 2020-03-24

**Authors:** Rodrigo P. Carvalho, Cleber F. N. Marchiori, Daniel Brandell, C. Moyses Araujo

**Affiliations:** ^1^ Materials Theory Division Department of Physics and Astronomy Ångström Laboratory Uppsala University Box 516 75120 Uppsala Sweden; ^2^ Department of Chemistry, Ångström Laboratory Uppsala University Box 538 75121 Uppsala Sweden

**Keywords:** batteries, density functional calculations, electrochemistry, electrode materials, organic cathodes

## Abstract

Several forms of organic materials have arisen as promising candidates for future active electrode materials for Li‐ion and post‐Li‐ion batteries, owing to a series of key features that encompasses sustainability, accessibility, and tunable electrochemical properties by molecular modifications. In this context, a series of organic electrode materials (OEMs) are investigated to further understand their thermodynamic and electronic properties. Through an evolutionary algorithm approach combined with first‐principles calculations, the crystal structure of lithiated and delithiated phases of these OEMs and their respective NO_2_‐substituted analogues are predicted. This framework allows a first assessment of their electrochemical and electronic properties and further understanding on the effects of the nitro group in the substituted compounds. NO_2_ is found to strongly affect structural and thermodynamic aspects during the electrochemical reaction with the reducing equivalents (Li^+^+e^−^), changing the OEM's character from a low‐potential anode to a high‐potential cathode by creating a localization of the additional electrons, thus resulting in a better‐defined redox‐active center and leading to a shift in the potential from 0.92 V to 2.66 V vs. Li/Li^+^.

## Graphical abstract

**From anodes to cathodes**: Organic materials have arisen as a promising alternative for the next generation of Li‐ion and post‐Li‐ion batteries. By following a state‐of‐the‐art theoretical framework, the crystal structure of a series of organic electrodes are predicted and their electronic and electrochemical properties are assessed. Moreover, organic anodes can be shifted to higher potential cathodes by insertion of NO_2_, presenting a pathway to design new organic cathode materials.

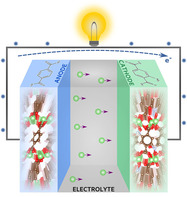

## Introduction

The world is currently undergoing a transition from a fossil fuel‐based economy to one based on the implementation of a more clean and renewable energy mix. Within this development, Li‐ion batteries are projected to take an even more prominent position as the primary electric energy storage device, which in turn will drastically increase the demand on the active redox materials used in the electrodes. In this context, it is problematic from a sustainability perspective that conventional Li‐ion battery technology is based on inorganic materials that are produced by energy intensive synthesis routes and involve extraction processes that can release toxic materials into the environment.^1^, ^2^ Furthermore, serious geo‐political and ethical issues are associated with the mining methods and ecology. Therefore, the development of greener and sustainable battery chemistry is very timely.^2^

Organic active electrode materials (OEMs) for Li‐ion batteries have arisen as promising alternatives by combining some important key features.^3^, ^4^ Firstly, they can be produced from abundant raw materials obtained from renewable resources, such as biomass. Secondly, the synthetic routes are highly versatile and generally operate at comparatively low temperatures. Thirdly, the potential chemical compositions involve large flexibilities, thereby rendering tunable properties that can meet end‐user‐specific demands. This is indeed a relevant feature that has the potential to give rise to significant breakthroughs in this technology field. Finally, the development of OEMs also opens up possibilities for easy end‐of‐life treatments and recycling routes, involving for instance combustion processes, which generate a closed materials lifecycle loop.^5^

Two main classes of organic electrode materials are known: they are either based on redox‐conducting polymers or on small redox‐active organic molecules (SOMs). Both categories face particular challenges in the development of suitable batteries. For SOMs, the major drawbacks are associated with (i) poor cycling stability, (ii) low electronic conductivity, and (iii) low energy density (especially volumetric).^6^, ^7^, ^8^, ^9^ The latter problem is mainly due to the fact that the functional redox groups need to be connected to conjugated organic cores, which in turn are not redox active but contributes to weight and volume. For SOMs, significant improvement on cycling stability has been achieved with the development of carboxylates^10^, ^11^, ^12^, ^13^, ^14^, ^15^, ^16^ following the pioneer work of Tarascon and co‐workers.^10^ Special focus has been given to dilithium terephthalate (Li_2_TP), because it can be straightforwardly obtained by recycling polyethylene terephthalate (PET) plastic and can deliver a reversible capacity of 300 mAh g^−1^.^10^ In these systems, the redox chemistry is controlled by the carbonyl groups, which tend to form stable enolates and display potentials around 1.0 V vs. Li/Li^+^ while the conjugated moiety acts as an electron reservoir.^2^ The low operating voltage renders them suitable for application as battery anode materials.

The development of organic active compounds with higher potentials for application as cathode materials is, in contrast, more challenging. Quinones and ketones are key functionalities in systems displaying the highest potentials of OEMs.^17^, ^18^, ^19^, ^20^ However, the potentials hardly exceed 2.5 V vs. Li/Li^+^, which limits the open circuit voltage of all‐organic cells to around 1.0–1.5 V. One promising compound that combines the stability of carboxylates with the higher potentials of quinones is dilithium oxyterephthalate.^21^ Through manipulation of the electronic structure of the organic moiety, Jouhara et al.^22^ achieved an all‐organic symmetric lithium‐ion cell displaying an output voltage of 2.5 V. One of the strategies for the realization of this electrode material involves substitution by Mg ions, which act as spectator cations in the host lattice. A small increase in the potential could also be obtained by stabilizing the *ortho* regioisomer. Another somewhat similar strategy is the use of substituents on the phenyl ring.^23^, ^24^ Different functional groups have been used to this end, such as amino, bromo, and nitro groups. However, only substitution by the latter caused a higher electrochemical potential.^23^

**Figure 1:**
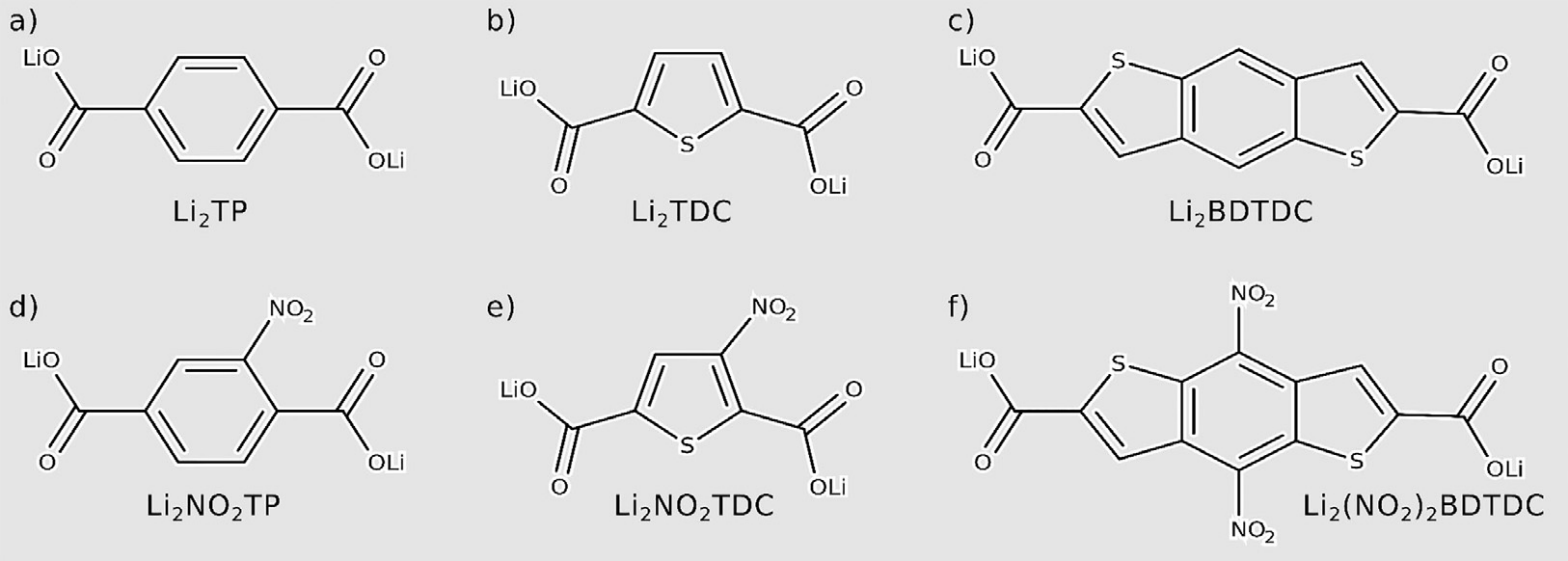
Lewis structures of the compounds: a) dilithium terephthalate (Li_2_TP); b) dilithium thiophenedicarboxylate (Li_2_TDC); c) dilithium benzodithiophenedicarboxylate (Li_2_BDTDC); d) dilithium nitroterephethalate (Li_2_NO_2_TP); e) dilithium nitrothiophenedicarboxylate (Li_2_NO_2_TDC); f) dilithium dinitrobenzodithionedicarboxylate (Li_2_(NO_2_)_2_BDTDC).

In the present study, we have employed an evolutionary algorithm combined with density functional theory (DFT) calculations to investigate the relationship between thermodynamics and structural/electronic property changes in the lithiation process of the following OEMs: dilithium terephthalate (Li_2_TP), dilithium thiophenedicarboxylate (Li_2_TDC), dilithium nitroterephthalate (Li_2_NO_2_TP), dilithium nitrothiophenedicarboxylate (Li_2_NO_2_TDC), dilithium benzodithiophenedicarboxylate (Li_2_BDTDC), and dilithium dinitrobenzodithiophenedicarboxylate [Li_2_(NO_2_)_2_BDTDC]. Through investigation of this series of structurally similar OEMs (Figure 1), the effects on the electrochemistry of two distinct modifications of the organic moieties can be systematically assessed, namely: i) the change of the conjugation length (Figures 1 a–c) and ii) the addition of redox‐active substituents (Figures 1 d–f). Among these compounds, Li_2_TP and Li_2_TDC have previously been investigated^25^, ^26^ by using similar methodology, and are here mainly used as reference systems. The calculated potentials range from 0.86 vs. Li/Li^+^ (Li_2_TP) to 2.66 vs. Li/Li^+^ (Li_2_(NO_2_)_2_BDTDC). For all studied systems, substitution by NO_2_ led to higher potentials, with the highest shift obtained for Li_2_(NO_2_)_2_BDTDC. A trend can be identified among the substituted compounds, where higher charge localization on the NO_2_ unit (and correspondingly less charge distributed on the organic ring) results in higher potentials. In fact, the redox‐active center is generally defined by the spatial localization of charges upon redox reactions.^27^ Thus, our results indicate that the introduction of well‐defined redox‐active centers, which are electronically decoupled from the conjugated part of the molecule, is a potentially useful way to achieve higher potential cathode materials.

## Results and Discussion

**Figure 2:**
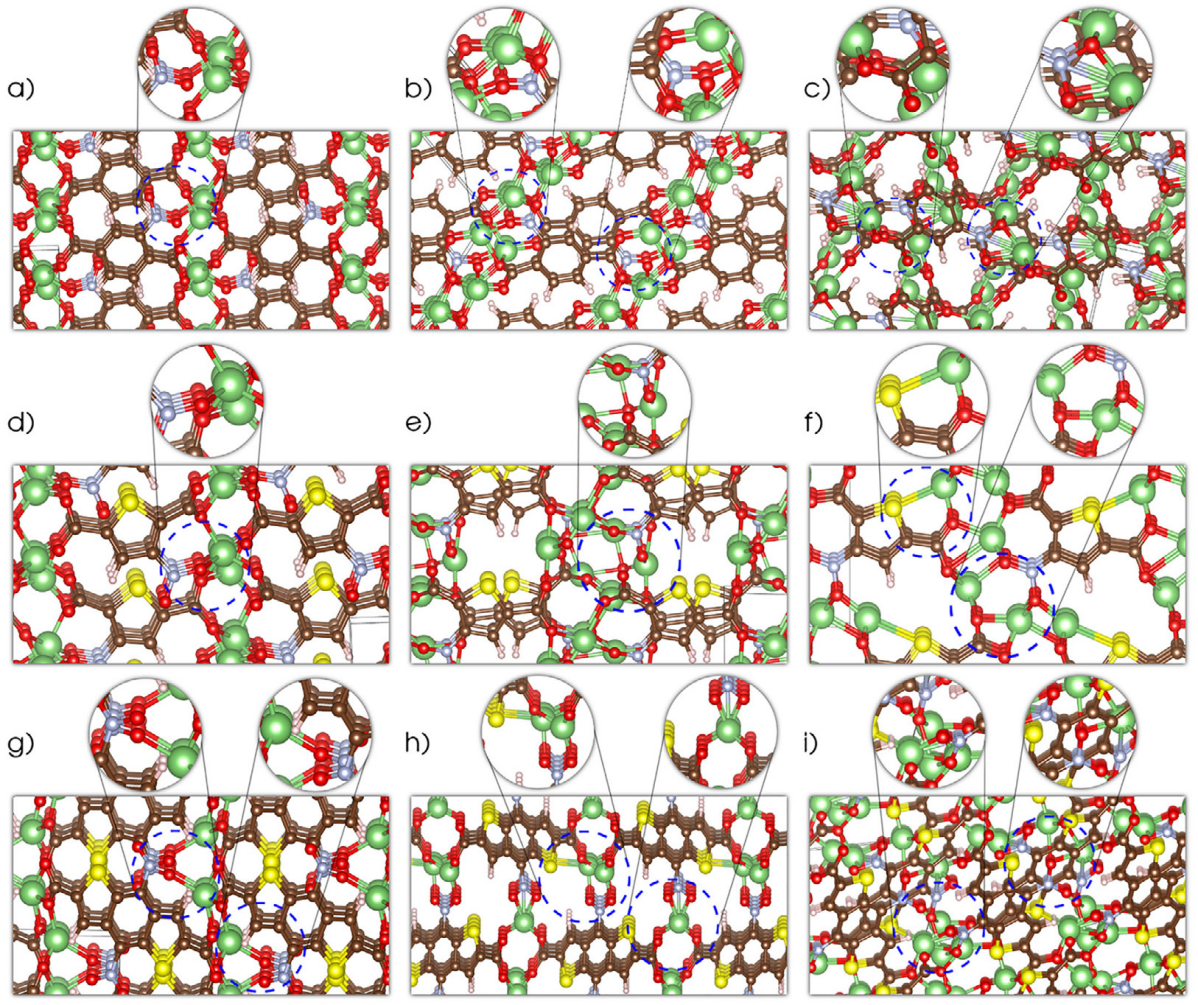
Predicted crystal structures: a) dilithium nitroterephthalate; b) trilithium nitroterephthalate; c) tetralithium nitroterephthalate; d) dilithium nitrothiophenedicarboxylate; e) trilithium nitrothiophenedicarboxylate; f) tetralithium nitrothiophenedicarboxylate; g) dilithium dinitrobenzodithiophenedicarboxylate; h) trilithium dinitrobenzodithiophenedicarboxylate; i) tetralithium dinitrobenzodithiophenedicarboxylate. The following color code applies for the atoms: red for oxygen, brown for carbon, yellow for sulfur, white for hydrogen, blue nitrogen, and green for lithium.

Determination of the crystal structures for OEM compounds involving both delithiated and lithiated phases is a challenging experimental task requiring the implementation of sophisticated operando spectroscopy and crystallography techniques.^12^, ^28^, ^29^ The alternative computational methodology applied here has been proven^26^, ^30^ to be able to predict such structures without experimental inputs, thereby allowing an understanding of the underlying electrochemistry at the atomic scale of such compounds. Figure 2 presents the resolved crystal structures for the NO_2_‐group containing Li_2_NO_2_TP, Li_2_NO_2_TDC and Li_2_(NO_2_)_2_BDTDC compounds in their delithiated and lithiated phases, including the insertion of up to two additional lithium ions. This corresponds to a theoretical capacity of 240 mAh g^−1^, 234 mAh g^−1^ and 141 mAh g^−1^, respectively, when considering the insertion of two lithium atoms, although one could expect the possibility of further inserting additional ions due to the number of available redox centers. For instance, the carbonyl and nitro groups are expected to accommodate one and two lithium ions, respectively,^23^ resulting in a maximum lithium uptake of four, four, and six ions for the Li_2_NO_2_TP, Li_2_NO_2_TDC, and Li_2_(NO_2_)_2_BDTDC materials, respectively. Thereafter, maximum theoretical capacities of 480, 468, and 423 mAh g^−1^ could be accessible for these structures, respectively.

All structures are generated in their respective energy minima by using the approach based on density functional theory interplayed with an evolutionary algorithm, as further described in the computational methods section. The structures of Li_2_TP, Li_2_TDC and Li_2_BDTDC are presented in the Supporting Information (Figure S1; corresponding CIF files are also available). All obtained parameters and corresponding space groups are shown in Tables S1, S2, and S3.

Starting with Li_2_NO_2_TP (Figures 2 a–c), one can observe that NO_2_ participates in the coordination of Li^+^ ions already in the delithiated phase, where otherwise Li^+^ is primarily coordinated by the carboxyl groups. The structure comprises Li‐rich layers interconnected with the organic moieties, which is similar to the TP structure (see the Supporting Information). As the compound undergoes further lithiation steps, the variation of the structure is small, with the main effect being an increase of the Li‐ion density in the “salt layer” of the structure.

The story is slightly different for Li_2_NO_2_TDC (Figures 2 d–f). The structure of the delithiated phase again contains Li‐rich layers, which here are interconnected by nitrothiophene units, and the NO_2_ groups again participate in the coordination of Li ions. Already during the first lithiation step, however, the Li‐rich layer displays a discontinuous density of Li ions; a conformation that persists upon the second lithiation step. Another important feature, which differs from the NO_2_‐free analogue Li_2_TDC, is the lack of coordination of Li ion by the S moiety. In contrast to the results obtained for Li_2_TDC (see the Supporting Information), NO_2_ and the carbonyl group dominate all cation coordination in Li_2_NO_2_TDC, whereas S does not contribute. S starts to coordinate one of the Li ions only after the second lithiation step. This is also consistent with the electronic structure obtained for these systems (discussed below). For Li_2_(NO_2_)_2_BDTDC (Figures 2 g–i), it can also be seen that the NO_2_ groups coordinate the Li ion in the delithiated phase. After the first lithiation step, however, the Li‐rich layer is broken up and the Li ions are coordinated by NO_2_ from one molecular unit and a carbonyl oxygen from another molecular unit. After the second lithiation, the Li‐rich layer reappears but with a clearly disordered structure. This result might well have an impact on the ionic transport in these materials (and thereby their rate performance), since the cation sites are not well connected.

**Figure 3:**
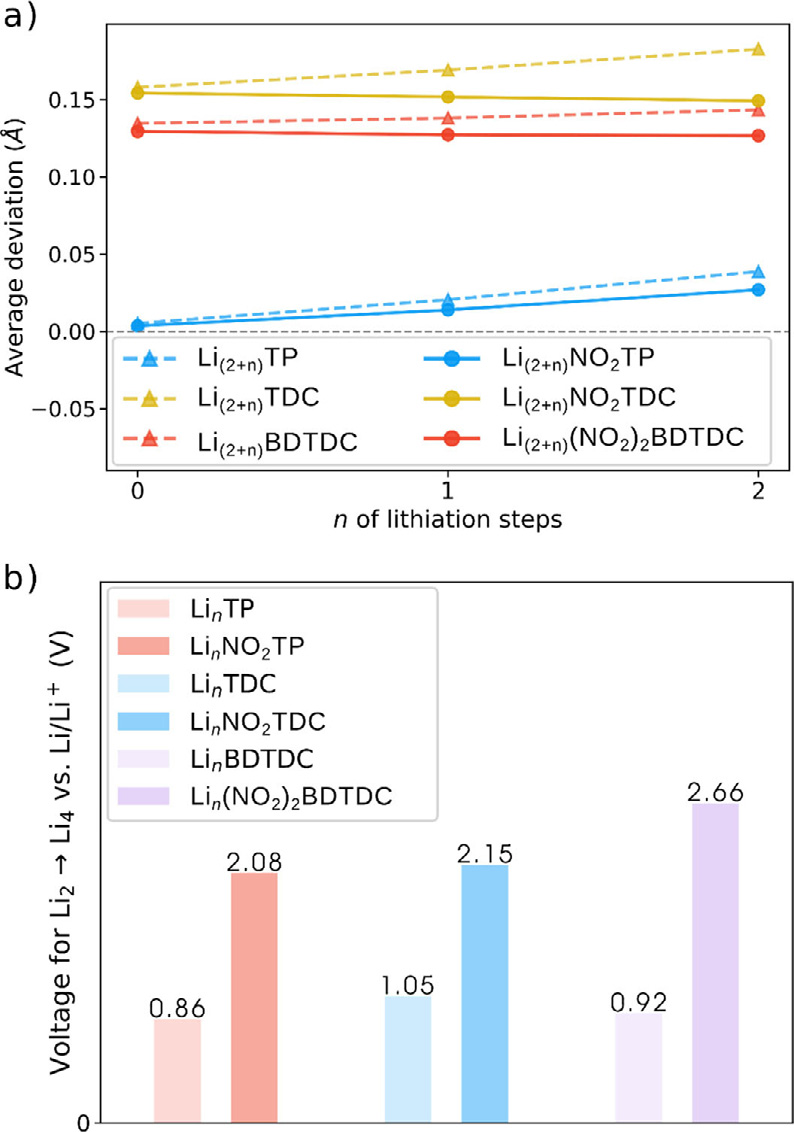
a) The average bond length deviation calculated by averaging the sum of absolute values of the differences between bond lengths and the mean bond length of the respective ring. b) Calculated average lithiation potentials for the two‐electron electrochemical reactions Li_2_(XDC)+2(Li^+^+e^−^)→Li_4_(XDC), where XDC represents the different organic dicarboxylates. The calculated potentials are referred to the potential of the Li/Li^+^ redox couple.

Figure 3 a shows the change in average bond length deviation (AD) upon lithiation for each compound. The AD is calculated by averaging the sum of absolute values of the differences between bond lengths and the mean bond length of the respective ring [see Equation (1)]. When AD is close to zero, the bond lengths around the ring are very similar, which is a signature of the benzene aromatic structure. The variation of AD upon lithiation indicates, quantitatively, geometric changes in the molecular ring when the system receives a new electron/lithium cation. Furthermore, it can be seen that the NO_2_ helps to prevent significant changes in the bond lengths of the ring structures upon lithiation in most of the compounds, which is due to the localization of the additional electrons in this unit, whereas less charge appears on the conjugated part of the molecules. This result is slightly different for Li_2_NO_2_TP than for the other NO_2_‐group‐containing compounds, because a significant amount of charge is still distributed over the ring.

The open circuit voltages [see Equation (2) and Figure 3 b] reveal that substitution with NO_2_ in all OEMs leads to a shift to higher potentials, where Li_2_(NO_2_)_2_BDTDC displays the highest value of 2.66 V vs. Li/Li^+^ upon insertion of two lithium atoms. Although this does not reveal the thermodynamic details of each lithiation step for these materials, such an estimated average potential can be considered suitable to represent the overall performance of each compound and the main electrochemical features induced by the functional group. One can thereby see that at proper combination of substituent and organic moiety, the potential could be shifted by about 1.8 V vs. Li/Li^+^. This is due to the tuning of the electronic structure, as discussed below.

**Figure 4:**
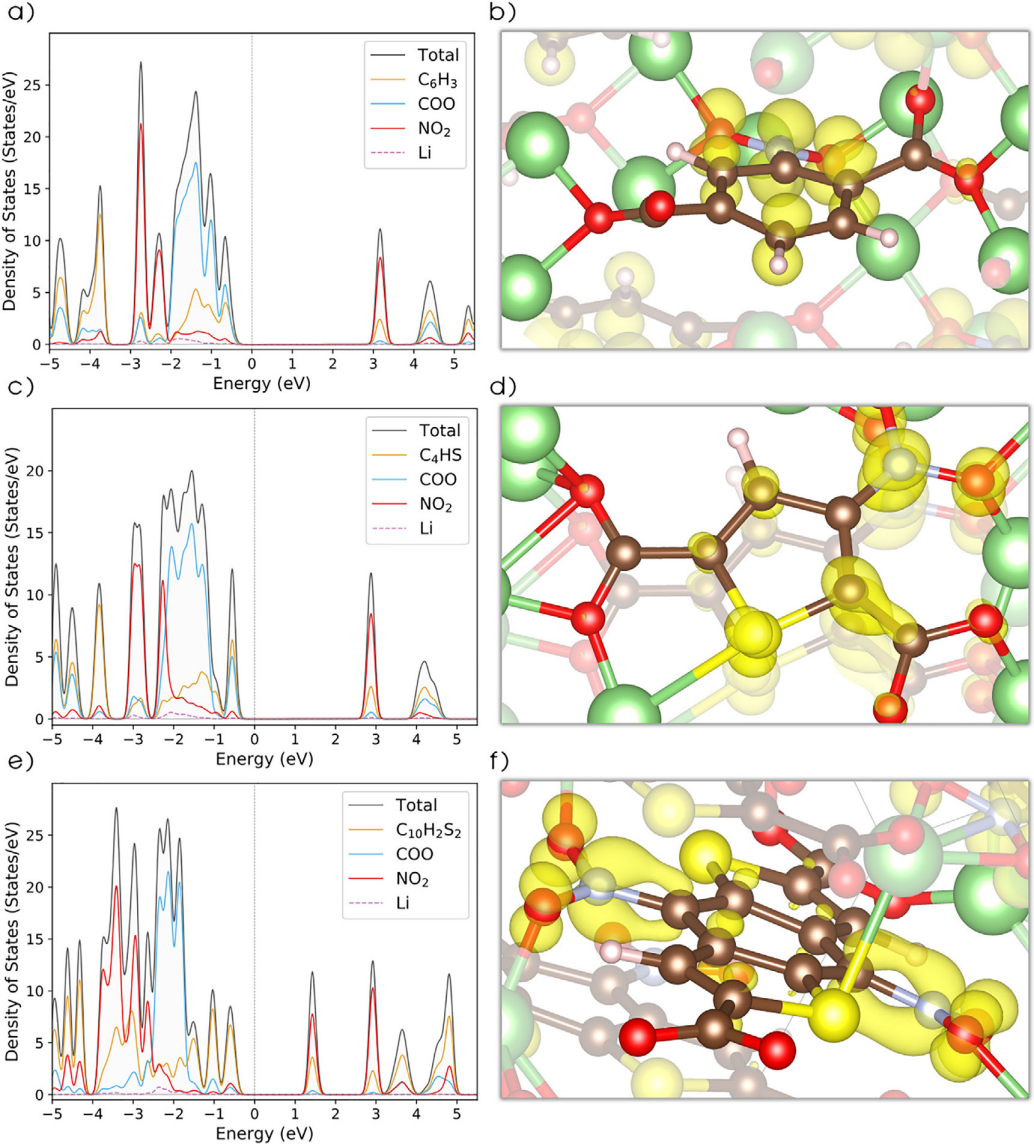
Density of States (DOS) and charge density isosurfaces (isosurface=0.01): a) Li_2_NO_2_TP and b) Li_4_NO_2_TP; c) Li_2_NO_2_TDC and d) Li_4_NO_2_TDC; e) Li_2_(NO_2_)_2_BDTDC and f) Li_4_(NO_2_)_2_BDTDC. Atom colors are as in Figure 2.

Figure 4 displays the total and projected density of states (DOS) for the Li_2_NO_2_TP (a), Li_2_NO_2_TDC (c) and Li_2_(NO_2_)_2_BDTDC (e) compounds. The first unoccupied band will mainly accommodate the additional electrons, which are inserted upon lithiation. The DOS has also been projected on different fragments of the compounds. The first unoccupied band is dominated by NO_2_ for all three compounds, with a small contribution from the organic ring and an even smaller participation from the carboxylate group, in clear contrast to the nonsubstituted compounds (without NO_2_ units), in which this first unoccupied band is composed of the carboxylate and the organic ring contributions (see Figure S2). The evolution of the DOS for the three lithiation phases (shown in Figure S3 for all OEMs) indicates that this NO_2_‐dominated band is indeed what becomes populated during the lithiation process. Furthermore, there is a delocalization of states in the DOS for the structures of Li_3_TP, Li_4_NO_2_TDC, and Li_3_BDTDC that could be related to a loss of symmetry upon the first lithiation step. Moreover, this is not recovered in the second lithiation step (see Table S1). The distribution of the received extra electrons after two lithiation steps for the three compounds are also shown in Figure 4 b, d, and f, where the localization on the NO_2_ units is obvious and most noticeable for the benzodithiophenedicarboxylate. Therefore, this localization could help to create a clearer redox‐active center than in the nonsubstituted compounds, which mainly delocalize these electrons over the carboxylate and the molecular ring (see Figure S4 for the electronic distributions). This, in turn, could be one of the reasons for the observed potential shift in Figure 3. It is then interesting to see that the TP and TDC (Figure 4 b and d, respectively) molecular units are still able to partially accommodate these additional electrons, acting as an electron reservoir, in contrast to BDTDC (Figure 4 f), which mainly localizes them on the NO_2_ unit.

**Figure 5:**
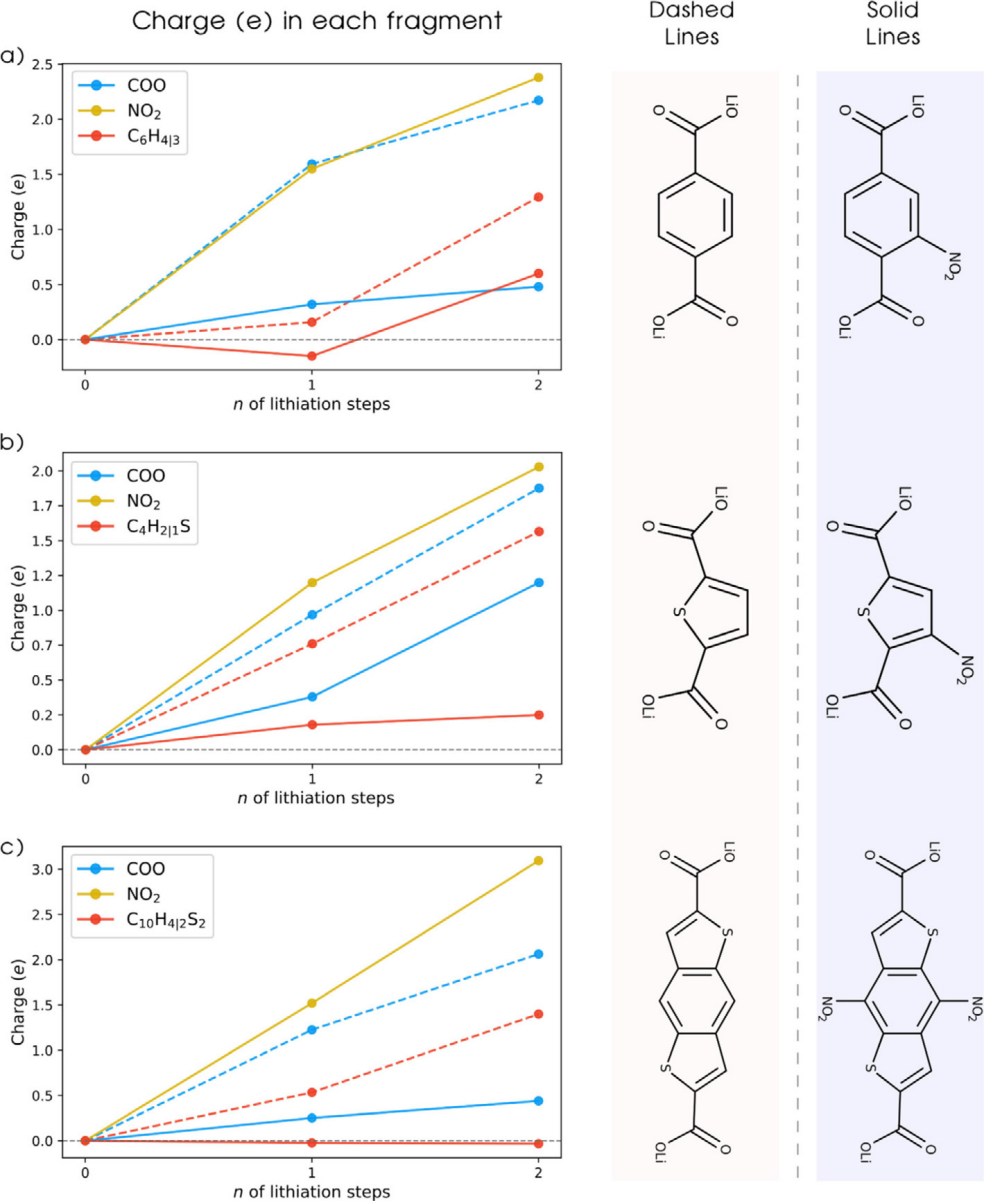
Bader charge analysis showing the charge variation in each molecular fragment normalized by their respective number of atoms. Dashed lines refer to nonsubstituted compounds and solid lines refer to the NO_2_‐substituted derivatives: a) NO_2_TP; b) NO_2_TDC; c) (NO_2_)_2_BDTDC.

A Bader charge analysis can shed light on the charge transfer upon lithiation, pinpointing the localization of charges in specific elements of the structure. The total (electronic) charge separated by fragments and subtracted from their respective amounts in the delithiated phase is shown in Figure 5. Thereby, we are only analyzing the distribution of the additional electrons on specific fragments following the lithiation process. For all compounds, the charge distribution changes drastically upon substitution of the nitro group, mainly shifting from the carboxylate and organic ring units for the nonsubstituted compounds (dashed lines), to the NO_2_ unit in the substituted compounds (solid lines). This correlates well with the results presented in Figure 4. Furthermore, the amount of charge in the molecular ring for NO_2_TP is still noticeable, in contrast to the values for NO_2_TDC and (NO_2_)_2_BDTDC, with the latter showing no significant changes upon lithiation. This could be the reason for the geometrical changes shown in Figure 3 a for NO_2_TP and the compounds without the nitro group (dashed lines), implying a clear correlation between structural changes of the organic ring units and the amount of charge, which they are able to accommodate. After the first lithiation step, NO_2_TP shows an unexpected charge loss on the ring unit followed by a larger charge gain in the next lithiation step. This happens for the nonsubstituted TP as well, thereby suggesting that a two‐electron/cation reaction could be more favorable to occur for the terephthalate, as suggested also in other works.^25^

This Bader charge analysis indicates a higher degree of localization of the charge on the potential redox units, dominated by the NO_2_ groups. This can be seen by comparing the solid and the dashed lines in Figure 5, where the nonsubstituted compounds show that a mixture of COO and the organic ring accommodate the charge. Such spatial localization of the additional electrons is consistent with the results shown in Figure 4 b, d, and f. In fact, these substitutions lead to a better definition of the redox‐active center in the molecules, which are likely useful when constructing cathode materials. Conversely, anode electrodes are expected to delocalize the electrons over the conjugated system, which instead is the case for all the nonsubstituted compounds TP, TDC, and BDTDC. These exhibit a clear delocalization of the additional electrons over the organic ring and the carboxylate units (see Figure S4). Therefore, this is a strong suggestion that the NO_2_ group changes the character of the compounds from low‐potential anodes to high‐potential cathodes by dominating the reduction process and creating a well‐defined redox‐active center.

## Conclusions

A theoretical approach comprising density functional theory and the employment of an evolutionary algorithm has been used to investigate the electrochemistry of the lithiation process of a range of NO_2_‐group‐containing compounds for organic electrode materials, and comparing them with their nonsubstituted analogues. We found that the Li coordination is dominated by carboxylate units in the delithiated nonsubstituted structures (Li_2_TP, Li_2_TDC and Li_2_BDTDC), with the S atom in TDC and BDTDC participating in the coordination already on the first lithiation step. For the delithiated structures with the nitro group, in contrast, Li ions are mainly coordinated by the carboxylate and NO_2_ units, with the latter showing a dominance of the coordination number upon lithiation of the structures. Additionally, S in NO_2_TDC and NO_2_BDTDC start to coordinate the Li first during the second lithiation step. The effect of the NO_2_ units is not limited to coordination of Li atoms, since the nitro group composes the first empty band of the electronic structure for all compounds. Thereby, they are responsible for accommodating the additional electrons absorbed upon the lithiation reaction, creating a spatial localization of charge that was not present in the nonsubstituted compounds and thus better defining the redox‐active center. As a consequence, the compounds undergo a shift to higher electrochemical potentials, which renders them adequate for use as cathode materials. It is thus seen that NO_2_BDTDC indeed could be a promising cathode material, displaying a potential of 2.66 V vs. Li/Li^+^ and a capacity of 141 mAh g^−1^. Moreover, the absence of drastic structural changes upon Li uptake suggests that these lithiation reactions are reversible for these electrodes, although further studies and experimental findings are required to fully elucidate these processes.

In light of these results, one can see how the presence of a substituent functional group can control the electrochemical behavior of an OEM and change both its structural and electronic properties. This could be a possible pathway to design new cathode materials by molecular engineering of the redox units and the electronic couplings throughout the organic moieties, by proper substitution of functional groups.

## Computational Methods

The crystal structure of each electrode and their lithiated phases were predicted by applying an evolutionary algorithm (EA) approach interplayed with density functional theory (DFT) calculations as implemented in USPEX^31^, ^32^, ^33^, ^34^ and VASP^35^, ^36^, ^37^ codes. The EA predicts each structure by evolving an initial population of randomly created structure‐candidates through a set of evolutionary operations,^32^, ^34^ building new generations of evolved structures towards the global minimum in the free energy landscape. The initial population is composed of 300–400 structures considering two molecular units per unit cell, from which a fraction of the best candidates—lowest energy after full geometry optimization with DFT—are selected together with new randomly generated structures to compose the next generation of 20–30 structures. Thereafter, this process is repeated consecutively to form new generations until a certain convergence criterion is achieved: the global energy minimum. For each generated structure, a full geometry optimization was performed in VASP using the projector‐augmented wave^36^ method with the GGA‐PBE^38^ as the exchange correlation functional in a multistep approach, increasing the plane‐wave cutoff energy (300, 300, 550, and 600 eV for each step) and the reciprocal‐space resolution (0.14, 0.12, 0.10, and 0.08 $2\pi {\overset{\circ }{A}}^{-1}$ for each step). Grimme's semi‐empirical corrections to the van der Waals interactions^39^ (DFT‐D2) were also considered, except for the Li atoms, since the increasing amount of this atom in the lithiated phases can generate an accumulated error due to fundamental limitations in DFT‐D theory.

Following the evolution process, the most suited candidate/structure of each material under study underwent a last geometry optimization with a higher plane‐wave cutoff energy of 600 eV and a finer gamma‐centered k‐mesh (6×6×6). Moreover, the HSE06^40^ hybrid functional was used (4×4×4 k‐mesh) for this final optimized crystal structure to achieve a better understanding of the thermodynamics and electronic structure of these systems. The importance of using this hybrid functional is to avoid the spurious electron's self‐interaction contribution present in the pure GGA scheme, which tends to overestimate the electron delocalization. Thereby, all results presented herein were obtained within this theoretical framework. Furthermore, a Bader^41^ charge analysis was performed for each system to understand the charge transfer in the system upon lithiation.

The average deviation (AD) of bond distances was calculated by averaging the sum of absolute values of the differences in bond distances between atoms that composed the structure (organic ring) and the mean bond length of the respective structure (ring), as shown by Equation (1):

(1) $$\bar{\Delta }=\frac{\sum_{i}^{n}\left|d_{i}-\bar{d}\right|}{n}$$

where *d*_i_ is a specific bond length, $\bar{d}$ is the mean bond length of the structure, and *n* is the number of bonds composing the structure. The open circuit voltage was calculated by using the same methodology presented in our previous publication,^26^ solving Equation (2):

(2) $$V\left(x\right)=-\frac{E\left({\mathrm{Li}}_{x_{1}}ℋ\right)-E\left({\mathrm{Li}}_{x_{0}}ℋ\right)-\left(x_{1}-x_{0}\right)E\left(\mathrm{Li}\right)}{x_{1}-x_{0}}$$

where ℋ is the electrode material being lithiated and *x*_0_ and *x*_1_ are the amount of initial and added Li, respectively.

## Conflict of interest

*The authors declare no conflict of interest*.

## Supporting information

As a service to our authors and readers, this journal provides supporting information supplied by the authors. Such materials are peer reviewed and may be re‐organized for online delivery, but are not copy‐edited or typeset. Technical support issues arising from supporting information (other than missing files) should be addressed to the authors.

Supplementary

## References

[1] G. Majeau-Bettez , T. R. Hawkins , A. H. Strømman , Environ. Sci. Technol. 2011, 45, 4548–4554. 21506538 10.1021/es103607c

[2] C. P. Grey , J. M. Tarascon , Nat. Mater. 2017, 16, 45–56. 10.1038/nmat4777 27994251

[3] T. B. Schon , B. T. McAllister , P.-F. Li , D. S. Seferos , Chem. Soc. Rev. 2016, 45, 6345–6404. 27273252 10.1039/c6cs00173d

[4] B. Häupler , A. Wild , U. S. Schubert , Adv. Energy Mater. 2015, 5, 1402034.

[5] S. Renault , D. Brandell , K. Edström , ChemSusChem 2014, 7, 2859–2867. 25170568 10.1002/cssc.201402440

[6] H. Chen , M. Armand , G. Demailly , F. Dolhem , P. Poizot , J. M. Tarascon , ChemSusChem 2008, 1, 348–355. 18605101 10.1002/cssc.200700161

[7] H. Chen , P. Poizot , F. Dolhem , N. I. Basir , O. Mentŕ , J. M. Tarascon , Electrochem. Solid-State Lett. 2009, 12, A102–A106.

[8] H. Chen , M. Armand , M. Courty , M. Jiang , C. P. Grey , F. Dolhem , J. M. Tarascon , P. Poizot , J. Am. Chem. Soc. 2009, 131, 8984–8988. 19476355 10.1021/ja9024897

[9] S. Renault , J. Geng , F. Dolhem , P. Poizot , Chem. Commun. 2011, 47, 2414–2416. 10.1039/c0cc04440g 21170429

[10] M. Armand , S. Grugeon , H. Vezin , S. Laruelle , P. Ribière , P. Poizot , J.-M. Tarascon , Nat. Mater. 2009, 8, 120. 19151701 10.1038/nmat2372

[11] Q. Zhao , C. Guo , Y. Lu , L. Liu , J. Liang , J. Chen , Ind. Eng. Chem. Res. 2016, 55, 5795–5804.

[12] H. H. Lee , Y. Park , K. H. Shin , K. T. Lee , S. Y. Hong , ACS Appl. Mater. Interfaces 2014, 6, 19118–19126. 25285535 10.1021/am505090p

[13] S. Renault , V. A. Oltean , C. M. Araujo , A. Grigoriev , K. Edström , D. Brandell , Chem. Mater. 2016, 28, 1920–1926.

[14] S. E. Burkhardt , J. Bois , J. M. Tarascon , R. G. Hennig , H. D. Abruña , Chem. Mater. 2013, 25, 132–141.

[15] X. Han , F. Yi , T. Sun , J. Sun , Electrochem. Commun. 2012, 25, 136–139.

[16] A. Iordache , D. Bresser , S. Solan , M. Retegan , M. Bardet , J. Skrzypski , L. Picard , L. Dubois , T. Gutel , Adv. Sustainable Syst. 2017, 1, 1600032.

[17] H. Alt , H. Binder , A. Köhling , G. Sandstede , Electrochim. Acta 1972, 17, 873–887.

[18] D. L. Williams , J. J. Byrne , J. S. Driscoll , J. Electrochem. Soc. 1969, 116, 2–4.

[19] A. Iordache , V. Maurel , J.-M. Mouesca , J. Pécaut , L. Dubois , T. Gutel , J. Power Sources 2014, 267, 553–559.

[20] Y. Liang , P. Zhang , J. Chen , Chem. Sci. 2013, 4, 1330–1337.

[21] B. Genorio , K. Pirnat , R. Cerc-Korosec , R. Dominko , M. Gaberscek , Angew. Chem. Int. Ed. 2010, 49, 7222–7224; 10.1002/anie.201001539 20803589

[22] A. Jouhara , N. Dupré , A.-C. Gaillot , D. Guyomard , F. Dolhem , P. Poizot , Nat. Commun. 2018, 9, 4401. 30353001 10.1038/s41467-018-06708-x PMC6199296

[23] Y. Park , D. Shin , S. H. Woo , N. S. Choi , K. H. Shin , S. M. Oh , K. T. Lee , S. Y. Hong , Adv. Mater. 2012, 24, 3562–3567. 22678780 10.1002/adma.201201205

[24] S. Renault , V. A. Oltean , M. Ebadi , K. Edström , D. Brandell , Solid State Ionics 2017, 307, 1–5.

[25] A. Banerjee , R. B. Araujo , M. Sjödin , R. Ahuja , Nano Energy 2018, 47, 301–308.

[26] C. F. N. Marchiori , D. Brandell , C. M. Araujo , J. Phys. Chem. C 2019, 123, 4691–4700.

[27] M. Saubanère , J.-S. Filhol , M.-L. Doublet in Physical Multiscale Modeling and Numerical Simulation of Electrochemical Devices for Energy Conversion and Storage (Eds.: A. A. Franco, M. L. Doublet, W. G. Bessler), Springer, Berlin, 2016, pp. 1–36.

[28] L. Fédèle , F. Sauvage , S. Gottis , C. Davoisne , E. Salager , J. N. Chotard , M. Becuwe , Chem. Mater. 2017, 29, 546–554.

[29] V. Medabalmi , R. Kothandaraman , Electrochim. Acta 2017, 232, 244–253.

[30] A. R. Oganov , C. J. Pickard , Q. Zhu , R. J. Needs , Nat. Rev. Mater. 2019, 4, 331–348.

[31] A. R. Oganov , C. W. Glass , J. Chem. Phys. 2006, 124, 244704. 16821993 10.1063/1.2210932

[32] A. R. Oganov , A. O. Lyakhov , M. Valle , Acc. Chem. Res. 2011, 44, 227–237. 21361336 10.1021/ar1001318

[33] A. O. Lyakhov , A. R. Oganov , H. T. Stokes , Q. Zhu , Comput. Phys. Commun. 2013, 184, 1172–1182.

[34] C. W. Glass , A. R. Oganov , N. Hansen , Comput. Phys. Commun. 2006, 175, 713–720.

[35] G. Kresse , J. Hafner , Phys. Rev. B 1993, 47, 558–561. 10.1103/physrevb.47.558 10004490

[36] G. Kresse , D. Joubert , Phys. Rev. B 1999, 59, 1758–1775.

[37] G. Kresse , J. Furthmüller , Phys. Rev. B 1996, 54, 11169–11186. 10.1103/physrevb.54.11169 9984901

[38] J. P. Perdew , K. Burke , M. Ernzerhof , Phys. Rev. Lett. 1996, 77, 3865. 10062328 10.1103/PhysRevLett.77.3865

[39] S. Grimme , J. Comput. Chem. 2006, 27, 1787–1799. 16955487 10.1002/jcc.20495

[40] J. Heyd , G. E. Scuseria , M. Ernzerhof , J. Chem. Phys. 2003, 118, 8207–8215.

[41] W. Tang , E. Sanville , G. Henkelman , J. Phys. Condens. Matter 2009, 21, 084204. 21817356 10.1088/0953-8984/21/8/084204

