# Supplementary material for: Tuning the Electrochemical Properties of Organic Battery Cathode Materials: Insights from Evolutionary Algorithm DFT Calculations
Source: ChemSusChem. 2020 Mar 24;13(9):2402–9. doi: 10.1002/cssc.201903450 (PMC7318659; doi:10.1002/cssc.201903450)
Supplement: Supplementary file 1 — Supplementary [file CSSC-13-2402-s001.pdf]

ChemSusChem

Supporting Information

Tuning the Electrochemical Properties of Organic Battery Cathode Materials: Insights from Evolutionary Algorithm DFT Calculations

Rodrigo P. Carvalho,[a] Cleber F. N. Marchiori,[b] Daniel Brandell,*[b] and C. Moyses Araujo*[a]

Table S1 Space group of all crystal structures for their de/lithiated phases.

	Electrodes					
**n of Li**	**TP**	**TDC**	**BDTDC**	**NO_2TP**	**NO_2TDC**	**$(NO_2)_2$BDTDC**
Li2	$P2_1/c$	$P2_1$	$P2_1/c$	$P2_1$	$P2_1$	$P2_1/c$
Li3	P1	$P2_1$	P1	$P\bar{1}$	P1	P1
Li4	P1	$P2_1$	$P2_1$	$P2_1$	P1	C2

Table S2 Lattice parameters of all crystal structures for their delithiated phases.

	Electrodes					
	**Li_2TP**	**Li_2TDC**	**Li_2BDTDC**	**Li_2NO_2TP**	**Li_2NO_2TDC**	**Li_2 $(NO_2)_2$BDTDC**
a (Å)	8.16	7.87	9.13	7.90	8.08	7.83
b (Å)	8.45	5.11	12.61	6.21	6.07	8.77
c (Å)	5.12	8.40	4.82	8.90	8.72	10.11
α (deg)	89.98	90.00	90.10	98.91	78.34	90.00
β (deg)	90.00	94.33	90.01	90.00	89.99	90.00
γ (deg)	85.17	90.00	86.77	90.00	89.99	73.51

Table S3 Volume in Å^3 of all crystal structures for their de/lithiated phases.

	Electrodes					
**n of Li**	**TP**	**TDC**	**BDTDC**	**NO_2TP**	**NO_2TDC**	**$(NO_2)_2$BDTDC**
Li2	351.64	337.25	553.76	431.76	418.62	665.31
Li3	327.75	336.21	539.45	426.91	383.58	729.86
Li4	371.31	336.60	550.10	428.78	377.39	654.28

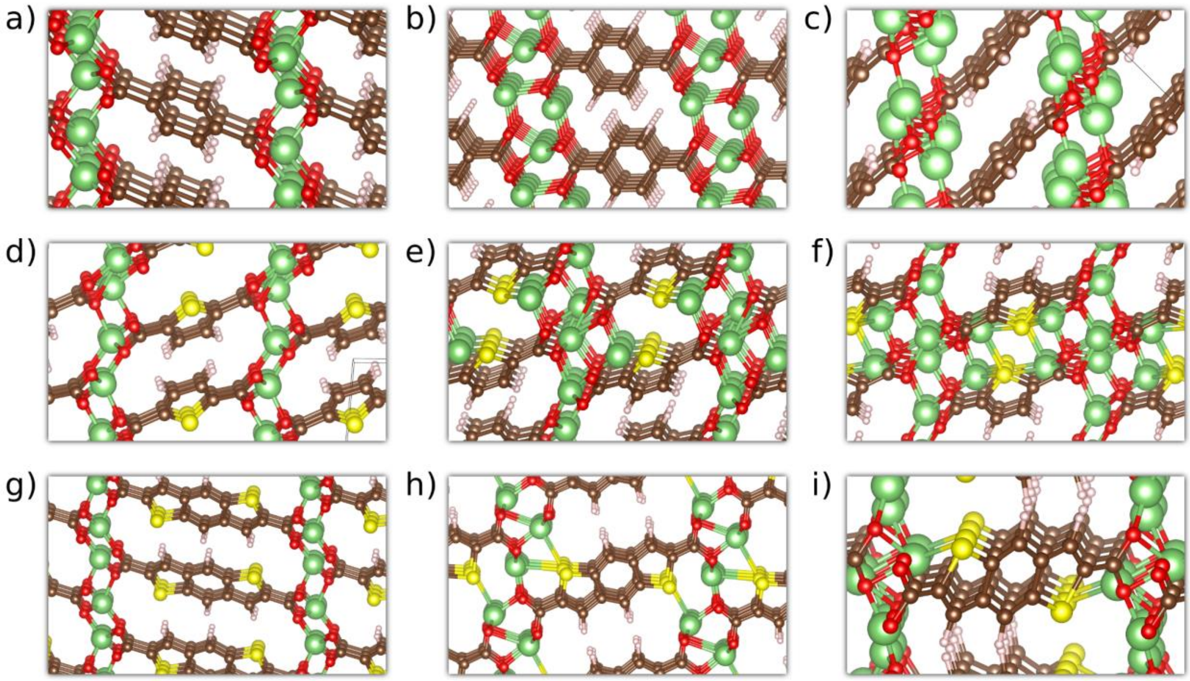

Figure S1 The predicted crystal structure for the de/lithiated phases for a) Li_2TP, b) Li_3TP, c) Li_4TP, d) Li_2TDC, e) Li_3TDC, f) Li_4TDC, g) Li_2BDTDC, h) Li_3BDTDC and i) Li_4BDTDC.

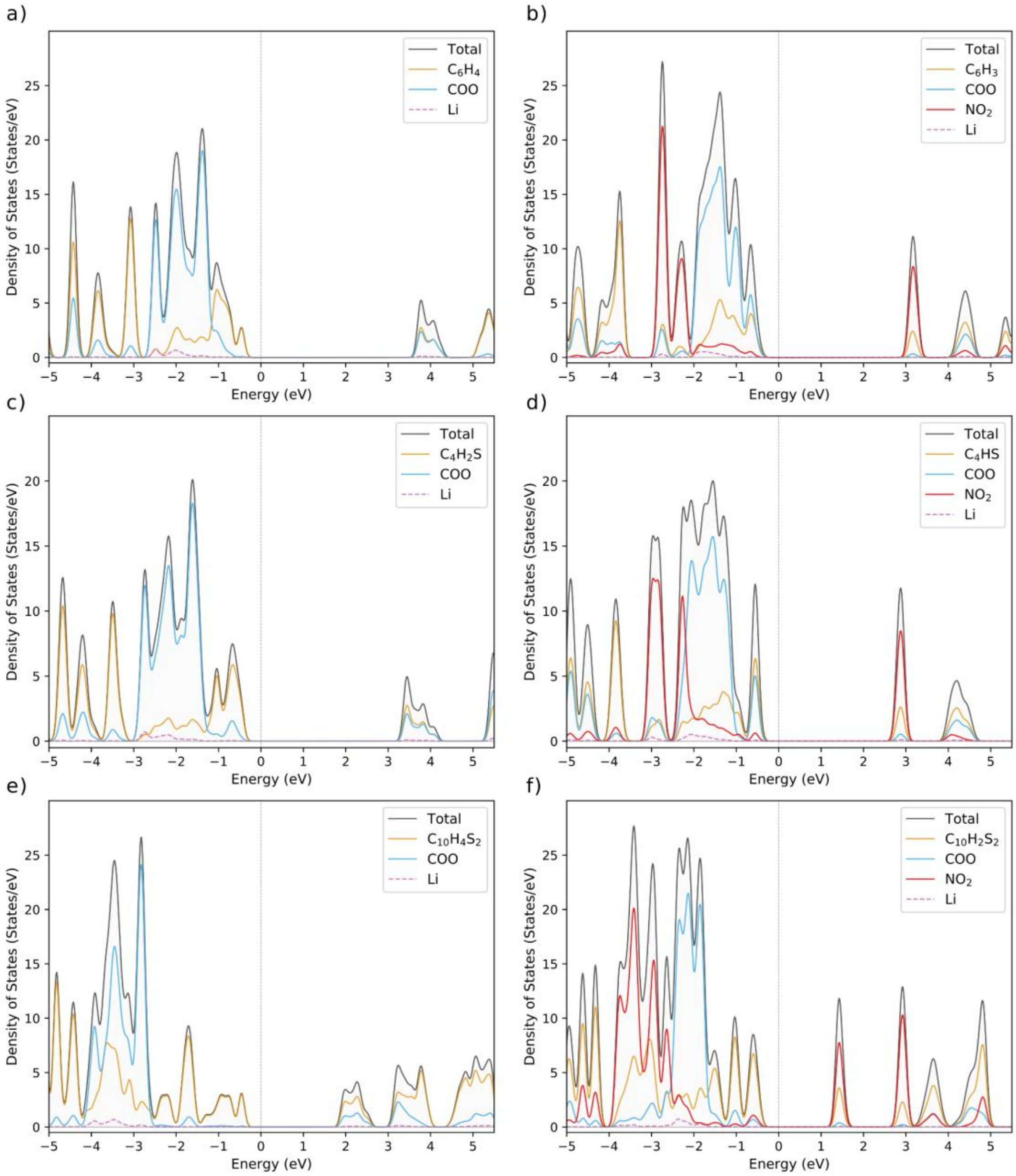

Figure S2 Total and fragment- projected density of states for a) Li_2TP, b) Li_2NO_2TP, c) Li_2TDC, d) Li_2NO_2TDC, e) Li_2BDTDC and f) $Li_2(NO_2)_2BDTDC$.

The fragment projected density of states on the Figure S2 shows how the NO_2 group overtakes the first unoccupied band over the carboxylate and the molecular ring units for all structures.

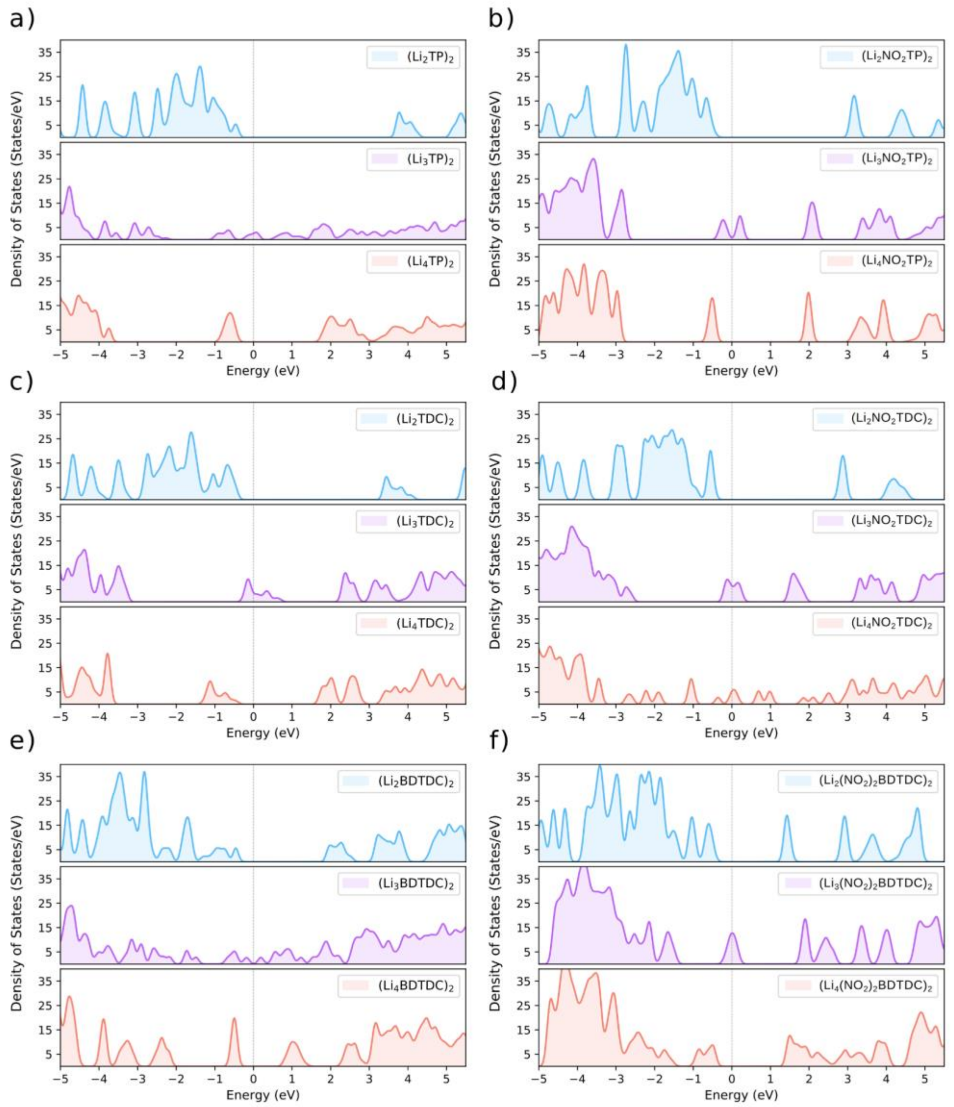

Figure S3 Evolution of DOS upon lithiation showing the population of the empty band for a) Li_2TP, b) Li_2NO_2TP, c) Li_2TDC, d) Li_2NO_2TDC, e) Li_2BDTDC and f) $Li_2(NO_2)_2BDTDC$.

The DOS evolution upon lithiation for all compounds. For some structures, one can note a delocalization of states that could be related to the loss of symmetry in the Li uptake process.

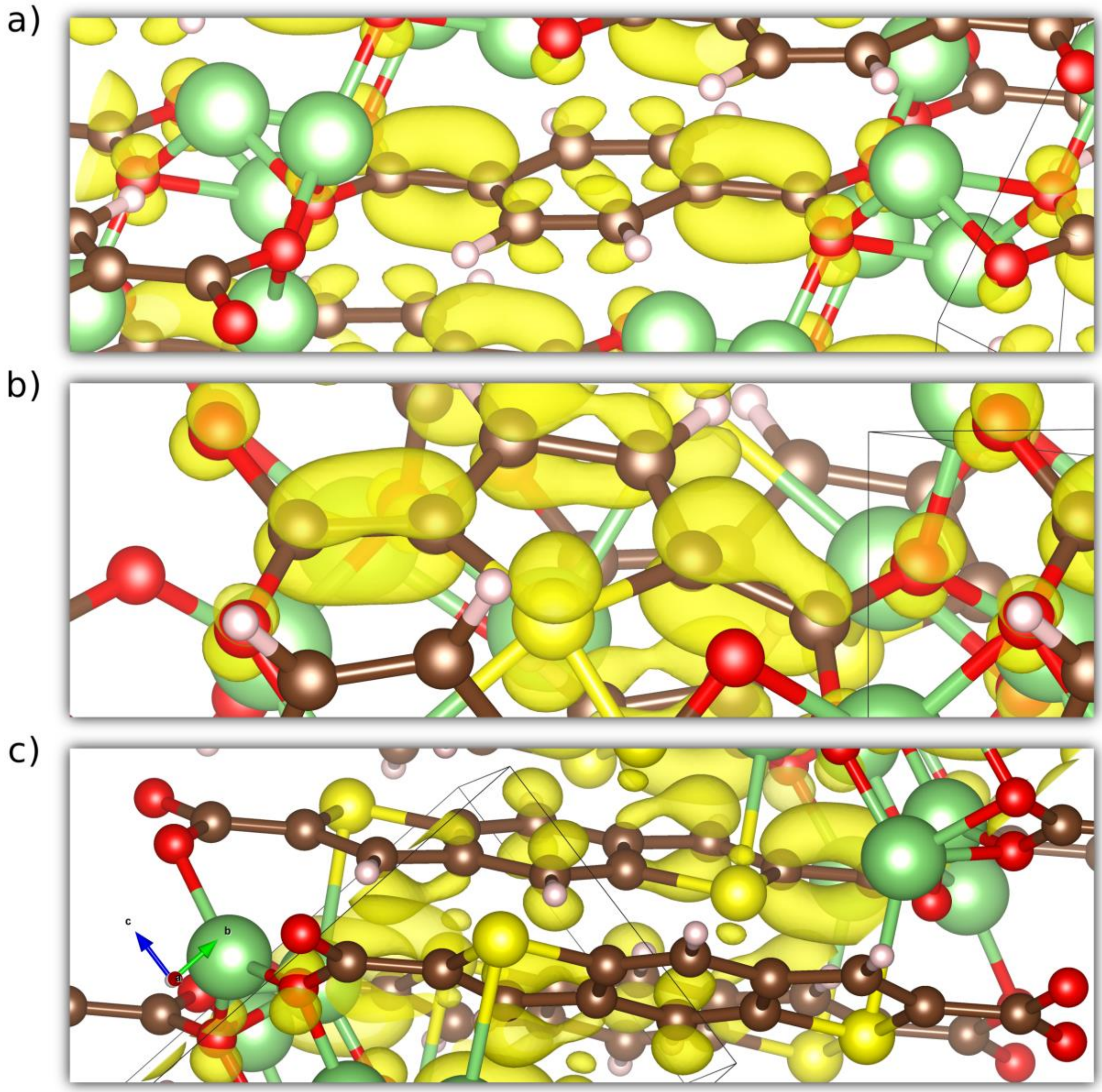

Figure S4 Partial charge density isosurface (isosurface = 0.01) for the extra added electrons on the lithiated phases for a) Li_4TP, b) Li_4TDC and c) Li_4BDTDC.

Figure S4 shows the charge density for the new received electrons on the second lithiation steps, pointing the clear delocalization of these electrons over the carboxylate and molecular ring units.
